# Real-Ambient Particulate Matter Exposure-Induced Cardiotoxicity in C57/B6 Mice

**DOI:** 10.3389/fphar.2020.00199

**Published:** 2020-03-31

**Authors:** Lianhua Cui, Limei Shi, Daochuan Li, Xiaobo Li, Xuan Su, Liping Chen, Qixiao Jiang, Menghui Jiang, Jing Luo, Andong Ji, Chen Chen, Jianxun Wang, JingLong Tang, Jingbo Pi, Rui Chen, Wen Chen, Rong Zhang, Yuxin Zheng

**Affiliations:** ^1^Department of Toxicology, School of Public Health, Qingdao University, Qingdao, China; ^2^Department of Toxicology, School of Public Health, Sun Yat-sen University, Guangzhou, China; ^3^Key Laboratory of Environmental Medicine Engineering, Ministry of Education, School of Public Health, Southeast University, Nanjing, China; ^4^Department of Toxicology, School of Public Health, Hebei Medical University, Shijiazhuang, China; ^5^School of Basic Medicine, Qingdao University, Qingdao, China; ^6^School of Public Health, China Medical University, Shenyang, China

**Keywords:** individual ventilated cages, real-life exposure, C57/B6 mouse, particulate matter, cardiotoxicity, molecular mechanism

## Abstract

It is generally accepted that exposure to particulate matter (PM) increases the risk of cardiovascular-related morbidity and mortality, though the exact mechanism behind this has yet to be elucidated. Oxidative stress plays a potentially important role in the mechanism of toxicity, with Nrf2 serving as a major antioxidant gene. In the current study, a Nrf2 knockout mouse model was used in combination with an individual ventilated cage (IVC)-based real-ambient PM exposure system to assess the potential cardiotoxicity induced by real-ambient PM exposure and the potential role of Nrf2 and related signaling in this endpoint. After 6- or 11-weeks exposure to PM, ICP-mass spectrometry was used to assess the metal depositions in the heart tissue following PM exposure. Functional and morphological changes in the hearts were investigated with echocardiography and histopathology, and oxidative stress levels were assessed with a serum malondialdehyde content assay. In the further mechanistic study, an RNA-seq technique was utilized to assess the gene transcription status in the hearts of C57/B6 mice exposed to PM with or without Nrf2 knockout. The expression levels of genes of interest were then further investigated with quantitative real-time PCR and western blotting. The results indicated that PM exposure resulted in significant elevation of sodium, potassium, selenium, and ferrum levels in mouse heart tissue. Meanwhile, significantly altered heart function and morphology were observed. Interestingly, Nrf2 knockout led to abolishment of PM-induced effects in several functional parameters but not the morphological changes. Meanwhile, elevated malondialdehyde content was observed in Nrf2 knockout animals. RNA-seq results revealed thousands of genes altered by PM exposure and/or Nrf2 knockout, and this affected several pathways, such as MAPK, phagosome, calcium signaling, and JAK-STAT. In subsequent molecular studies, enhanced nuclear translocation of Nrf2 was also observed following PM exposure, while the MAPK signaling pathway along with related JAK-STAT and TGF-β1 pathway genes, such as p38MAPK, AKT, TAK1, JAK1, STAT3, GRB2, TGFb1, and SMAD2, were confirmed to be affected by PM exposure and/or Nrf2 knockout. The data suggested that PM may induce cardiotoxicity in C57/B6 mice in which Nrf2 plays both protective and detrimental roles involving cardiac-related pathways, such as MAPK, JAK-STAT, and TGF-β1.

## Introduction

Exposure to particulate matter (PM) is an environmental risk factor for human health. PM is derived from a variety of resources: coal combustions, diesel exhausts, and biomass burning, etc. (Gour et al., 2018; Gadi et al., 2019). The relatively small size of PMs, especially the PMs with a diameter smaller than 2.5 um (PM2.5) allows them to be inhaled into deeper respiratory tracts, even into the circulatory system, and to induce detrimental health effects within the cardiopulmonary system (Brook et al., 2004; Almendra et al., 2017). While developed countries, such as the United States and Japan, displayed acceptable levels of ambient PM, some areas of the world displayed relatively high levels, far beyond the healthy levels set by the World Health Organization (Puett et al., 2011; Zhou et al., 2015; Gorai et al., 2018; Khan et al., 2018), posing a significant threat to human health. PM exposure has been associated with cardiovascular diseases (Ensor et al., 2013; Sohn et al., 2016) and cardiovascular-related mortality (Zhang C. et al., 2018; Hvidtfeldt et al., 2019). While part of the mechanism, such as increased risk of myocardial infarction, has been identified, much still remains to be elucidated. Further investigation is urgently needed to facilitate a better understanding of PM-induced cardiotoxicity and risk assessment.

Epidemiological studies of PM exposure have provided us with the most relevant data regarding the human health effects of PM exposure. However, such studies are often limited by challenges when it comes to determining exact exposure doses and the variances associated with large population studies, and this makes it difficult to perform mechanistic studies or draw clear mechanistic conclusions (Brook et al., 2010; Yang et al., 2016). Laboratory studies of PM indeed revealed molecular mechanisms of PM-induced toxicities, but most studies used PM particles that were collected and washed from filters (Kundu and Stone, 2014; Liu T. et al., 2018) and exposed animals with either intratracheal instillation or inhalation chambers (Chen et al., 2013; Pardo et al., 2015). Loss of the water-soluble partition of the PMs and relatively high doses of exposure over a short period of time decreased the relevancy of such studies. To represent the real-life situation to the best extent, the individual ventilated cages (IVC) real-life exposure system was utilized in the current study, and it almost completely mimics the real-life situation of PM exposure in accordance with the outdoor atmospheric PM pollution (Lawal, 2017). The exposure doses and PM compositions were also recorded accurately, allowing for better interpretation of the results, thus providing valuable data for risk assessments.

One major proposed mechanism of toxicity for the PMs was oxidative stress; this was supported by several studies in which PM exposure-induced reactive oxygen species (ROS) generation and oxidative stress in the cardiovascular system were observed (Mills et al., 2009; Brook et al., 2010; Lawal, 2017). On the other hand, nuclear factor (erythroid-derived 2)-like 2 (Nrf2) had been considered as a major antioxidant response gene, regulating the expression of a series of antioxidant genes (Zhang et al., 2015; Lawal, 2017) that are believed to counteract the ROS generation and oxidative stress, including the ones induced by PM exposure (Deng et al., 2013). To investigate the role of Nrf2, Nrf2 knockout mice and wildtype littermate C57/B6 mice were utilized. With the help of Nrf2 knockout mice model, the roles of Nrf2 in PM-induced cardiotoxicity were further elucidated.

This study utilized the IVC real-life exposure system to investigate the cardiovascular effects of PM exposure in C57/B6 mice, the roles of Nrf2 with knockout mice, and gene expression alterations following PM exposure, with or without Nrf2 knockout. Our data added to the knowledge base of PM-induced toxicity and provided mechanistic evidence for the management of PM pollution.

## Materials and Methods

### Materials

PCR primers were designed and synthesized by Sangon (Shanghai, China). The reverse transcription kit and PCR master mix were purchased from Yisheng (Shanghai, China). Antibodies were purchased from Abcam (Cambridge, MA, US) and Cell signaling Technology (Danvers, MA, US). All other chemicals used in this study were purchased from Sigma-Aldrich (Shanghai, China) and were of the highest grade obtainable.

### Animal Grouping and Housing

The Nrf2 knockout mice and corresponding wildtype control mice (C57B6/J) (6–8 weeks) were obtained from the animal lab of School of Public Health, China Medical University, which has proved to be a successful model for the Nrf2 functional investigation (Sun et al., 2018). Upon arrival, the animals were adapted for 24 h and genotyped; wildtype littermate mice were then randomly assigned to wildtype-control (WTC) or wildtype-exposure (WTE) groups, while the knockout mice were randomly assigned to knockout-control (KOC) or knockout-exposed (KOE) groups. Please refer to Supplementary Figure 1 for genotyping results. The mice were then kept under standard housing conditions (12-h lighting/dark cycle, 22–24°C room temperature, and water and food were provided *ad libitum*). All the procedures used in this study have been approved by the Qingdao University Animal Care and Use Committee in keeping with the National Institutes of Health guidelines.

### IVC Exposure

The IVC exposure method was as described in Li et al. (2019). Briefly, a unique system was built with both exposure and control chambers. The conditions in both chambers were identical: temperature (20–25°C), humidity (40–60%), pressure (15–20 Pa), ventilation frequency (18–20/h), air-flow rate (0.17 m/s), and noise (30–35 dB). The exposure chamber was ventilated with unfiltered air, while the control chamber was ventilated with HEPA-filtered air. Animals were housed in ventilation cages (five in each cage) and exposed to unfiltered air or filtered air for 24 h/day for 7 days/week with *ad libitum* access to food and water. The PM concentration in the chambers were measured with an Aerosol Detector DUSTTRAKTM II and analyzed with an Aerodynamic Particle Sizer Spectrometer 3321 (TSI Incorporated, Shoreview, MN, USA). The cumulative lung burden was calculated: cumulative burden = MV × T × CON × DF. MV: minute ventilation (mL/min); T: total exposure time (min); CON: mean concentration (mg/m^3^); DF: pulmonary deposition fraction (m^3^).

### Echocardiography

Upon desired timepoints, the animals were anesthetized with 80 mg/kg sodium pentabarbiturate via intraperitoneal injection, placed on a warm plate, and then the probe of the transducer was gently placed on the left side of the sternum between the fourth and sixth ribs. M-mode images were then captured at the papillary muscle level. Each image loop included 10 to 20 cardiac cycles. Data were averaged from at least three cycles per loop. The left ventricular end-diastolic dimension (LVEDD) and left ventricular end-systolic dimension (LVESD) were directly measured, while other parameters, such as the left ventricular end-diastolic volume (LVEDV), left ventricular end-systolic volume (LVESV), ejection fraction (EF), fractional shortening (FS), stroke volume (SV), and cardiac output (CO), were derived automatically by the Vevo 2,100 imaging system (Visual Sonics, Toronto, ON, Canada). Please refer to Supplementary Figure 2 for representative echocardiography pictures.

### Sample Collection

Upon desired timepoints, the animals were anesthetized with 80 mg/kg sodium pentabarbiturate via intraperitoneal injection and sacrificed. The serum, heart, lung, liver, fat tissue, spleen, and kidney were collected. Tissues for histological assessments were fixed in 4% formaldehyde in phosphate-buffered saline, while other tissues were archived in −80°C freezer until further use.

### ICP Mass

Four heart tissue samples from each 11-weeks group (WTC, WTE, KOC, and KOE) were randomly selected and subjected to ICP Mass spectrometry (Agilent 7500CX, CA, US) for the detection of metals. Briefly, the 0.2 g samples were digested by adding 6 mL nitric acid and 1 mL hydrogen peroxide and heated to 200 degrees Celsius for 30 min. The resulting solution was calibrated to 10 mL with ultrapure water and then subjected to ICP-Mass. The parameters used were high-salt nebulizer, quartz nebulization chamber, quartz glass rods, nebulization temperature: 2 degree Celsius, RF power 1,500 W, Carrier gas 1.25 L/min, Sample depth 7.5 mm, helium 5 mL/min, and Flow rate (sample) 15 mL/min. The metal concentrations in the samples were calculated with an equation: Concentration (mg/kg) = Acquired reading (ng/mL) × 10 / sample mass (g) x 1,000. The limit of detection was 0.005 mg/kg, while the limit of quantification was 0.01 mg/kg. Blank samples and standards were included in each batch of samples. The variation between two independent measurements on the same sample was below 10%.

### Histological Assessment

After 24-h fixation in 4% formaldehyde, the hearts were cut at 60% height counting from the apex, processed, and embedded in paraffin as described in Lv et al. (2018). Cross-sections of the hearts were made with a Leica RM2160 microtome at 6 μm thickness. Sections were then deparaffinized and stained with hematoxylin and eosin (Beyotime, Beijing, China) according to the protocol provided by manufacturer. Pictures of the sections were taken with a OlympusBX59, and then ImageJ (NIH, US) was used to assess the thickness of the right ventricular wall as described in Jiang et al. (2012). Please refer to Supplementary Figure 3 for the quantification method for right ventricular wall thickness.

### RNA-Seq Assay

Four heart tissue samples from each 6-weeks group (WTC, WTE, KOC, and KOE) were randomly selected and subjected to RNA-seq assay. The whole assay, including the RNA extraction, was performed by Novogene (Shanghai, China). Briefly, sample RNA degradation and contamination were checked on 1% agarose gels by visually confirming the sharpness of the RNA bands and that they were free from non-RNA contaminants. RNA purity was checked using the Nano Photometer spectrophotometer (IMPLEN, CA, USA). RNA concentration was measured with Qubit® RNA Assay Kit in Qubit 2.0 Flurometer (Life Technologies, CA, USA). RNA integrity was assessed with the RNA Nano 6000 Assay Kit of the Bioanalyzer 2100 system (Agilent Technologies, CA, USA). A total of 3 μg RNA per sample was used for generating the sequencing libraries with NEBNext UltraTM RNA Library Prep Kit for Illumina® (NEB, USA) following manufacturer's recommendations. The clustering of the index-coded samples was performed on a cBot Cluster Generation System using TruSeq PE Cluster Kit v3-cBot-HS (Illumia) according to the manufacturer's instructions. After cluster generation, the library preparations were sequenced on an Illumina Hiseq platform and 125 bp/150 bp paired-end reads were generated. Clean data (clean reads) were obtained by removing reads containing adapter, reads containing ploy-N, and low-quality reads from raw data. Meanwhile, the Q20, Q30, and GC content of the clean data was calculated. All the downstream analyses were based on the clean data with high quality. Reference genome and gene model annotation files were downloaded from the genome website directly. The index of the reference genome was built using Hisat2 v2.0.5, and paired-end clean reads were aligned with the reference genome using Hisat2 v2.0.5. Hisat2 was selected as the mapping tool. Feature Counts v1.5.0-p3 was used to count the reads numbers mapped to each gene, and then FPKM of each gene was calculated based on the length of the gene and the read counts mapped to this gene. A differential expression analysis of two conditions/groups (two biological replicates per condition) was performed using the DESeq2 R package (1.16.1). DESeq2 provides statistical routines for determining differential expression in digital gene expression data using a model based on the negative binomial distribution. The resulting *P*-values were adjusted using the Benjamini and Hochberg's approach for controlling the false discovery rate. Genes with an adjusted *P* < 0.05 found by DESeq2 were assigned as differentially expressed. Cluster Profiler R package was used to test the statistical enrichment of differential expression genes in KEGG pathways.

### qRT-PCR

mRNA was extracted from the archived 6-weeks heart tissues with the Trizol agent (Yisheng, Shanghai, China) according to the manufacturer-provided protocol. The concentration and purity were determined with a Nanodrop One (Thermo Scientific, Waltham, Massachusetts, US), and then quantitative RT-PCR was performed with an CFX-96 PCR machine (Bio-Rad, Hercules, California, US). Briefly, the reactions were imitated by incubating the samples at 95°C for 30 s, and this was then followed by 40 cycles of extension (95°C, 5 s and then 60°C, 30 s). The relative expression fold changes relative to WTC samples were quantified with the delta-delta Ct method. Three independent samples were assessed per group.

### Western Blotting

Achieved 6-weeks heart samples were homogenized in a RIPA buffer (Beyotime, Beijing, China) with a 1:100 PMSF (Beyotime, Beijing, China) and 1:100 phosphatase inhibitor cocktail (Epizyme, Shanghai, China), which was added for 30 min, and then centrifuged at 14,000 g for 10 min. The protein concentrations of the samples were determined with a BCA kit (Beyotime, Beijing, China) following instructions from the manufacturers, and then standard SDS-PAGE electrophoresis was performed. To confirm the activation level of Nrf2, an additional batch of samples was processed with the nuclear and cytoplasmic extraction kit (Epizyme, Shanghai, China). Western blotting was then performed for Nrf2 in both the cytoplasmic and nuclear protein of the same samples. Proteins were transferred to the PVDF membrane and probed with primary antibodies (SMAD2, TAK1, and pTAK1 from Abcam; pSMAD2, p38-MAPK, p-p38MAPK, and Nrf2 from Cell Signaling Technology; and ATP1a1 from Affinity; the dilution ratios were 1:1,000 for all the primary antibodies; internal control was GAPDH from Bioss, and it had a dilution ratio of 1:5,000) and secondary antibodies (Epizyme goat anti rabbit/mouse IgG at 1:5,000). The bands were visualized with a Fusion Solo S (Vilber Lourmat, Collégien, France) and quantified with ImageJ (NIH, US) software. Three independent samples were assessed per group.

### Malondialdehyde (MDA) Assay

The 6-weeks serum samples were subjected to a commercially available kit (Solarbio, Beijing, China, BC0025) for MDA concentration. Briefly, 100 ul serum was mixed with a 300 ul MDA assay working solution and 100 ul MDA assay detection solution and then incubated at 100 degree Celsius for 1 h. The resulting solutions were centrifuged at 14,000 g for 10 min, 200 ul of supernatants were then moved to 96-well plates, and the absorbance was read at 450, 532, and 600 nm with a microplate reader (Thermo Varioskan LUX, Waltham, Massachusetts, US). The concentration of MDA in serum was calculated:

$$\begin{array}{l} \text{MDA}(\text{nmol/mL})=(12.9\times (\Delta \text{A}532-\Delta \text{A}600)-2.58\\ \times \Delta \text{A}450)\times \text{Vtotal/Vsample} \end{array}$$

### Statistical Analysis

Data were presented as mean ± standard derivation (SD). Statistical analysis was performed with SPSS 17.0. A factorial design analysis of variance (ANOVA) was used to assess differences among groups. Results were considered statistically significant when *P* < 0.05.

## Results

### General Parameters

The general parameters of animals (body weight, heart weight, heart index, liver weight, liver index, and total mortality after 11-weeks treatment) are reported in Table 1. At 6 weeks of treatment, liver weight (both absolute weight and relative weight) seemed to be affected slightly. No other significant changes were observed. At 11 weeks, significant changes in both heart and liver weight were observed, while no remarkable changes were observed in whole body weight. The highest mortality was observed in the KOE group animals (16.667%).

**Table 1:** General parameters of the animals at 6 or 11 weeks.

	**WTC**	**WTE**	**KOC**	**KOE**
**Six-week**, ***N*** **=** **8 per group**				
Body weight (g)	18.696 ± 1.221	19.979 ± 2.049	19.481 ± 1.816	19.686 ± 1.027
Heart weight (g)	0.112 ± 0.009	0.119 ± 0.004	0.115 ± 0.016	0.113 ± 0.007
Relative heart weight (%)	0.604 ± 0.059	0.601 ± 0.075	0.595 ± 0.104	0.573 ± 0.046
Liver weight (g)	0.978 ± 0.079^a^	1.114 ± 0.064^a^^b^	0.876 ± 0.164^b^^c^	0.986 ± 0.0860^b^^c^
Relative liver weight (%)	5.228 ± 0.241^a^	5.642 ± 0.825^b^	4.472 ± 0.510^a^^b^	5.015 ± 0.420^b^
**Eleven-week**, ***N*** **=** **24 per group**				
Body weight (g)	24.310 ± 1.996	24.150 ± 2.444	23.860 ± 2.400	23.380 ± 1.476
Heart weight (g)	0.132 ± 0.017	0.138 ± 0.025^a^	0.129 ± 0.017	0.124 ± 0.017^a^
Relative heart weight (%)	0.543 ± 0.055	0.572 ± 0.087^a^	0.542 ± 0.052	0.530 ± 0.073^a^
Liver weight (g)	1.125 ± 0.165	1.158 ± 0.201^a^	1.055 ± 0.179^a^	1.025 ± 0.156^a^
Relative liver weight (%)	4.618 ± 0.490	4.786 ± 0.607^a^	4.402 ± 0.445^a^	4.373 ± 0.535^a^
Total mortality (%)	0	9.091%	4.546%	16.667%

*WTC, wild type control; WTE, wild type exposure; KOC, knock out control; KOE, knock out exposure; BW, Body weights; OW, organ weights; BW-OW, remaining body weight after removing organ. Data are means ± SD*.

a *significantly different from WTC group, different letter represent significance between groups at p < 0.05*.

b *significantly different from WTE group, different letter represent significance between groups at p < 0.05*.

c *significantly different from KOC group, different letter represent significance between groups at p < 0.05*.

### PM Composition and Exposure Dose

The size and composition of the PM particles were reported in our previous study (Li et al., 2019). Briefly, during the 11-weeks PM exposure in Shijiazhuang, 2017, the PM concentration were constantly monitored in the ambient air of the study site as well as inside the chambers. The results indicated that the mean daily PM2.5 concentration in ambient air during weeks 1–6 and weeks 1–11 was 132.58 and 138.1 μg/m^3^, respectively. The corresponding mean concentration of PM2.5 in the exposure chambers was 79.98 and 86.5 μg/m^3^, respectively. The calculated cumulative lung burden of PM for the mice was 39.24 and 77.82 μg/mouse, respectively.

In addition to the exposure concentration and already published PM composition data, ICP-mass spectrometry was performed on the mice hearts. The major metal elements (Na, Mg, Ni, Cu, Al, K, Zn, Se, Ca, Cr, Sr, Ba, Mn, Fe, and Pb) were assessed, and the results are reported in Table 2. Since no significant differences were observed between wildtype and knockout animals receiving same treatment (clean air or PM exposure), samples were pooled to demonstrate the deposition of metals following PM exposure. For separated knockout animal data, please refer to Supplementary Table 1. Significant changes in PM exposed animals relative to control were observed in the levels of Na, K, Se, and Fe.

**Table 2:** The analysis of cardiac metals contents.

	**Control (mg/kg)**	**Exposure (mg/kg)**	***p***
Na	1609.730 ± 79.969	1473.909 ± 84.421	0.005^*^
Mg	184.108 ± 15.892	191.128 ± 11.412	0.327
Ni	0.064 ± 0.035	0.205 ± 0.165	0.034^*^
Cu	6.269 ± 0.377	6.306 ± 0.536	0.874
Al	27.674 ± 11.361	29.875 ± 9.454	0.680
K	2325.631 ± 155.823	2547.081 ± 143.440	0.010^*^
Zn	21.377 ± 2.249	23.326 ± 2.552	0.127
Se	0.289 ± 0.073	0.360 ± 0.038	0.028^*^
Ca	42.986 ± 7.617	48.390 ± 19.583	0.479
Cr	0.783 ± 0.418	1.083 ± 0.232	0.097
Sr	0.364 ± 0.080	0.405 ± 0.093	0.366
Ba	0.765 ± 0.173	0.889 ± 0.214	0.224
Mn	0.573 ± 0.064	0.634 ± 0.056	0.061
Fe	84.691 ± 11.127	97.351 ± 8.101	0.021^*^
Pb	0.167 ± 0.047	0.174 ± 0.060	0.796

* *Statistically different between the two groups (P < 0.05)*.

### Echocardiography Results

**Figure 1:**
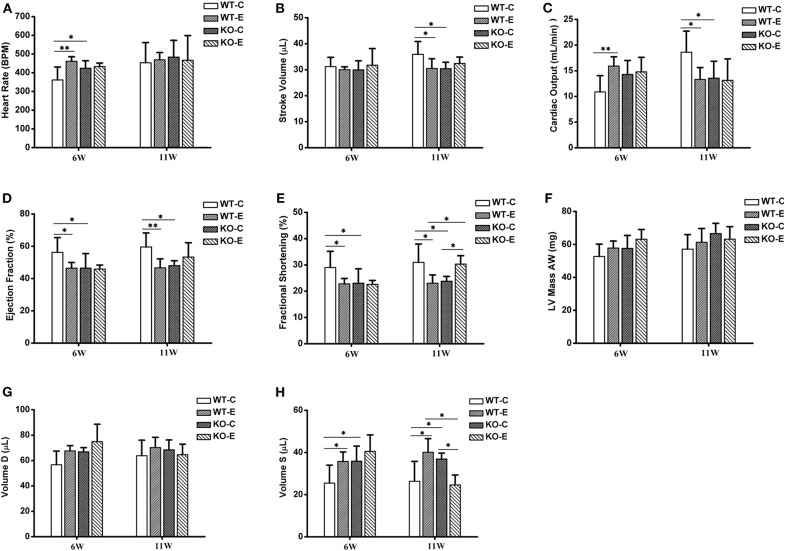
Echocardiography results. For 6 or 11 weeks, 6–8-weeks C57/B6 mice, with or without Nrf2 knockout, were exposed to either filtered air or air containing particulate matter (PM). The animals were then anesthetized with 80 mg/kg pentobarbital via intraperitoneal injection and subjected to a Vevo 2,100 imaging system for echocardiography. *N* = 4–5 per group. WT-C, wildtype control; WT-E, wildtype exposed. KO-C, knockout control; KO-E, knockout exposed. *Statistically different between the two groups (*P* < 0.05). **Statistically different between the two groups (*P* < 0.01). **(A)** Quantitative results for heart rate. **(B)** Quantitative results for stroke volume. **(C)** Quantitative results for cardiac output. **(D)** Quantitative results for ejection fraction. **(E)** Quantitative results for fractional shortening. **(F)** Quantitative results for LV mass AW. **(G)** Quantitative results for Volume D. **(H)** Quantitative results for Volume S.

Echocardiographic parameters with significant changes were reported in Figure 1. At 6 weeks of exposure, the heart rate was significantly elevated in WTE and KOC groups relative to the WTC group, while no remarkable changes were observed at 11 weeks of exposure (Figure 1A). No significant changes were observed in stroke volume after 6 weeks of exposure, while significant decreases were observed in WTE and KOC groups animals after 11 weeks of exposure (Figure 1B). Cardiac output gave the most interesting data: animals in the WTE group first had significantly elevated cardiac output after 6 weeks of exposure and then had remarkably decreased cardiac output after 11 weeks of exposure (Figure 1C). Ejection fraction results indicated significant decrease in WTE and KOC groups at both 6 and 11 weeks (Figure 1D). Decreased fractional shortening was observed in the WTE and KOC groups at both 6 and 11 weeks, while the KOE group significantly increased relative to the WTE or KOC group at 11 weeks (Figure 1E). LV mass data indicated a trend of elevation in KOE group mice at 6 weeks but not statistically significant (Figure 1F). Similar results were also observed for diastolic volume data (Figure 1G). For the systolic volume data, WTE and KOC animals both exhibited higher systolic volume at 6 and 11 weeks, while KOE animals had a remarkably decrease relative to WTE and KOC at 11 weeks (Figure 1H).

### Histopathological Assessments

**Figure 2:**
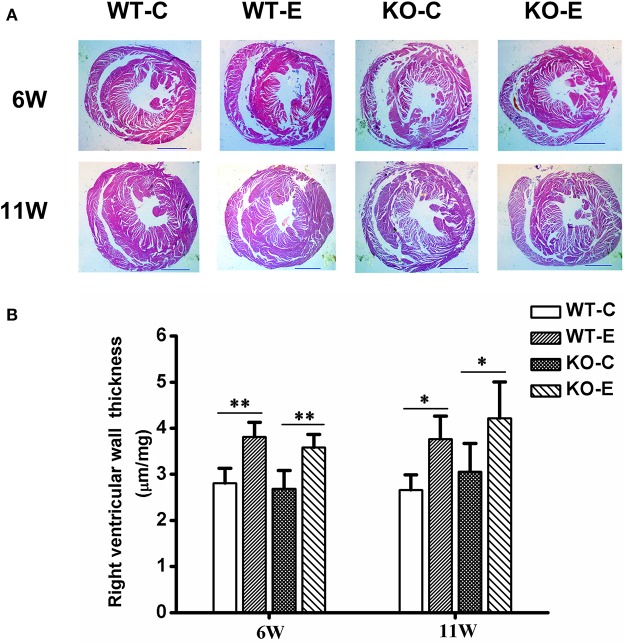
Histopathology results. For 6 or 11 weeks, 6–8-weeks C57/B6 mice, with or without Nrf2 knockout, were exposed to either filtered air or air containing particulate matter (PM), and the hearts were then collected, fixed in 4% buffered formaldehyde for 24 h, and then histologically processed for hematoxylin and eosin staining. The right ventricular wall thickness was then measured and analyzed. *N* = 3–4 per group. WT-C, wildtype control; WT-E, wildtype exposed; KO-C, knockout control; KO-E, knockout exposed. **(A)** Representative hematoxylin and eosin stained sections for WT-C, WT-E, KO-C, and KO-E groups after 6- or 11-weeks treatment. Scale bars represent 1,000 um. **(B)** Quantification of the right ventricular wall thickness. *Statistically different between the two groups (*P* < 0.05). **Statistically different between the two groups (*P* < 0.01).

After 6-weeks or 11-weeks exposure, histopathological assessments revealed a significantly thickened right ventricular wall in the WTE group relative to the WTC group and those in the KOE group relative to KOC group, but no significant interaction was observed between knockout and exposure (Figure 2). For the method of right ventricular wall thickness measurement, please refer to Supplemental Materials.

### Effects of PM Exposure and/or Nrf2 Knockout in the Heart Transcriptome

**Figure 3:**
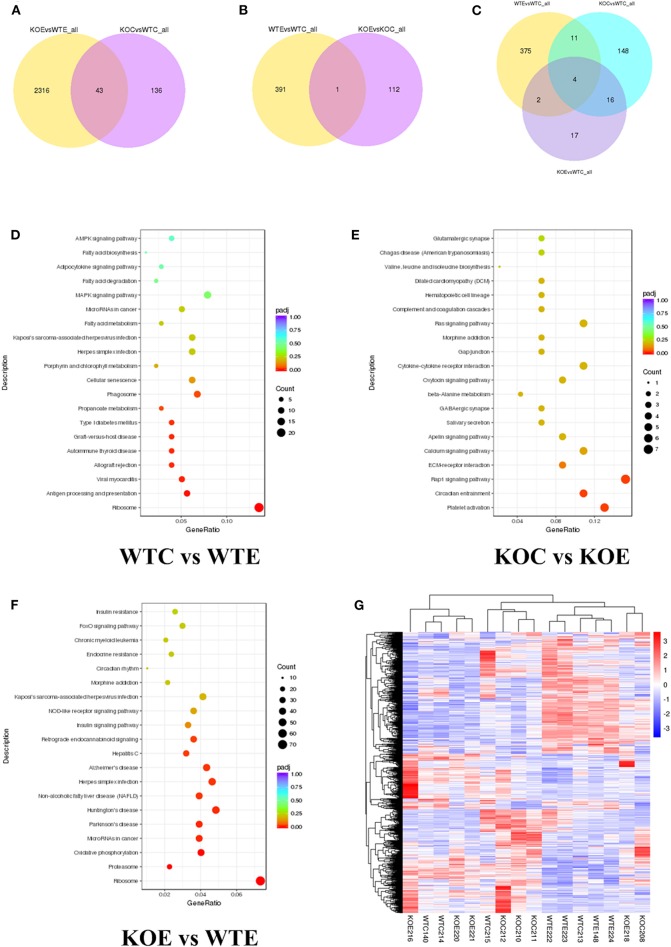
RNA-seq results. For 6 weeks, 6–8-weeks C57/B6 mice, with or without Nrf2 knockout, were exposed to either filtered air or air containing particulate matter (PM), and the hearts were collected, snap-frozen in liquid nitrogen, and subjected to RNA-seq assay. *N* = 4 per group. WTC, wildtype control; WTE, wildtype exposed; KOC, knockout control; KOE, knockout exposed. **(A)** Venn diagram showing the number of differentially expressed genes (DEGs) shared by KOE vs. WTE and KOC vs. WTC. **(B)** Venn diagram showing the number of DEGs shared by WTE vs. WTC and KOE vs. KOC. **(C)** Venn diagram showing the number of DEGs shared by WTE vs. WTC, KOC vs. WTC, and KOE vs. WTC. **(D)** Pathway enrichment graph for WTC vs. WTE. **(E)** Pathway enrichment graph for KOC vs. KOE. **(F)** Pathway enrichment graph for KOE vs. WTE. **(G)** Heatmap for all the samples.

RNA-seq results revealed thousands of genes altered by PM exposure and/or Nrf2 knockout. The number of differently expressed genes (DEGs) among different treatment groups are reported in Figure 3. The effects of knockout of Nrf2, with or without the presence of PM2.5 were analyzed: comparing to wildtype control animals, 179 DEGs were identified in knockout control animals. Meanwhile, 2359 DEGs were identified in knockout PM-exposed animals relative to wildtype PM-exposed animals. Of these, 43 DEGs were recognized as shared by the two comparison pairs (Figure 3A). To determine the effects of PM2.5 exposure, with or without Nrf2 knockout, the DEGs between wildtype control animals/wildtype PM-exposed animals and DEGs between knockout control animals/knockout PM-exposed animals were analyzed. A total of 392 DEGs were identified for the former and 113 DEGs were for the latter. Only one gene is shared between the two pairs (Figure 3B). To comprehensively assess the interactions between PM2.5 exposure and Nrf2 knockout, DEGs were identified among several pairs: wildtype control and wildtype PM-exposed; wildtype control and knockout control; and wildtype control and knockout PM-exposed. The results indicated 392, 179, and 32 DEGs, respectively. Among all the identified DEGs, four of them were shared among all the comparison pairs. The DEG-based KEGG pathway enrichment results are reported in Figures 3D–F. Major pathways observed with remarkable changes included the MAPK, phagosome, calcium, and Ras signaling pathways. The most affected pathways following PM exposure in wildtype animals include the MAPK signaling pathway and the phagosome pathway, while the calcium signaling pathway and the Ras signaling pathway seem to be most affected in Nrf2 knockout animals in response to PM exposure. Comparing the PM-exposed wildtype animals with PM-exposed Nrf2 knockout animals revealed that oxidative phosphorylation and the insulin signaling pathway may be differently altered under the stress of PM exposure. The heatmaps of all samples are reported in Figure 3G. Additionally, the top 20 upregulated or downregulated genes among the groups are attached in Supplementary Table 2.

### qRT-PCR Results

**Figure 4:**
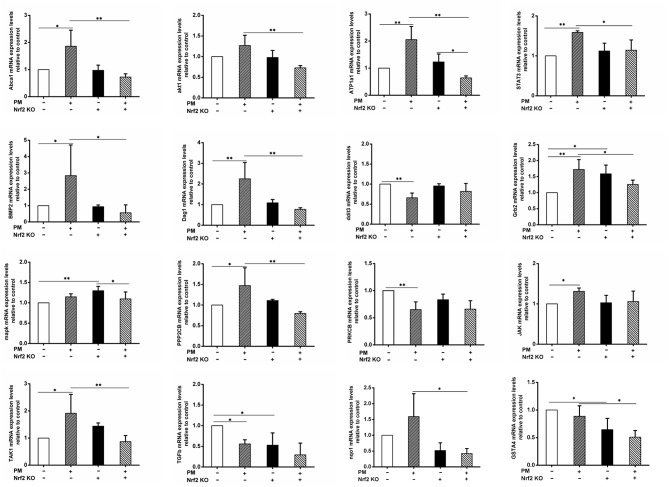
qRT-PCR results. For 6 weeks, 6–8-weeks C57/B6 mice, with or without Nrf2 knockout, were exposed to either filtered air or air containing particulate matter (PM), and then the hearts were collected, snap-frozen in liquid nitrogen, and subjected to quantitative reverse transcription PCR. Primers used included Abca1, akt1, ATP1a1, STAT3, BMP2, Dag1, ddit3, Grb2, MAPK, PPP2CB, PRKCB, TAK1, TGFb, and JAK. *N* = 3 per group from three independent experiments. *Statistically different between the two groups (*P* < 0.05). **Statistically different between the two groups (*P* < 0.01).

qRT-PCR was performed to confirm the mRNA level changes of selected DEGs detected in the RNA-seq assay. The primers used are reported in Table 3. The results indicated results that were similar to those seen in the RNA-seq assay (Figure 4). Among the 14 genes tested, the expression levels of ABCA1, BMP2, PPP2CB, DAG1, TAK1, JAK, Grb2, and STAT3 exhibited similar changes: PM exposure in wildtype animals significantly elevated the gene expression levels, while the Nrf2 knockout seemed to have effectively decreased the expression levels relative to the wildtype exposed animals (WTE group). ATP1A1 also displayed similar changes, with an additional significant decrease of the KOE group expression relative to the KOC group. The expression level of AKT1 did not significant differ between the WTC and WTE groups, but KOE group still had remarkably lower expression levels comparing to the WTE group. DDIT3 and PRKCB exhibited a similar pattern: they were decreased by PM exposure in wildtype animals, while Nrf2 knockout seemed to negate such changes. Regarding the expression levels of MAPK, no changes were observed in wildtype animals. While Nrf2 knockout increased the expression levels without the presence of PM, PM exposure along with Nrf2 knockout decreased the MAPK expression relative to the KOC animals. Additionally, the expression levels of TGF-β1 were significantly decreased in WTE and KOC animals relative to the WTC group, with a lower average value in the KOE group (not statistically significant). Finally, the expression levels of two Nrf2 target genes, Nqo1 and GSTA4, in the KOE group both significantly decreased relative to WTE group with an additional significant decrease in KOC group relative to WTC group for GSTA4.

**Table 3:** Primer sequences used in qRT-PCR.

**Primer**	**Sequence**
NFE2L2-Forward	CAGCCATGACTGATTTAAGCAG
NFE2L2-Reverse	CAGCTGCTTGTTTTCGGTATTA
PRKCB-Forward	CAAGTCTGCTGCTTTGTTGTAC
PRKCB-Reverse	TCTTAAACTTGTGTTTGCTCCG
PPP2CB-Forward	GCTTTTATGATGAGTGCCTACG
PPP2CB-Reverse	GGTCCAGTGTATCTATGGATGG
AKT1-Foeward	TGCACAAACGAGGGGAATATAT
AKT1-Reverse	CGTTCCTTGTAGCCAATAAAGG
MAPK1-Forward	ATCTCAACAAAGTTCGAGTTGC
MAPK1-Reverse	GTCTGAAGCGCAGTAAGATTTT
DDIT3-Forward	CTCCAGATTCCAGTCAGAGTTC
DDIT3-Reverse	ACTCTGTTTCCGTTTCCTAGTT
ATP1A1-Foeward	GAAGAAATCCATCGCTTACACC
ATP1A1-Reverse	GTTCACAAGTTTGTCCGTTTTG
Tak1-Forward	CCCTTCAATGGAGGAAATTGTG
Tak1-Reverse	CTCCAAGCGTTTAATAGTGTCG
TGFbeta1-Forward	CCAGATCCTGTCCAAACTAAGG
TGFbeta1-Reverse	CTCTTTAGCATAGTAGTCCGCT
BMP2-Forward	AGTAGTTTCCAGCACCGAATTA
BMP2-Reverse	CACTAACCTGGTGTCCAATAGT
Dag1-Forward	CTCCTTGAACCAGAATAGCGTC
Dag1-Reverse	ATAACCAAGTTGGGCAGACATA
Abca1-Forward	CCTCAGAGAAAACAGAAAACCG
Abca1-Reverse	CTTTGCTATGATCTGCACGTAC
JAK-Forward	ACATTCTTACCAAAGTGCGTTC
JAK-Reverse	GCTGAATGAATCTGCGAAATCT
STAT3-Forward	TGTCAGATCACATGGGCTAAAT
STAT3-Reverse	GGTCGATGATATTGTCTAGCCA
Grb2-Forward	ATAAGGCAGAACTCAATGGGAA
Grb2-Reverse	ACATCATTTCCAAACTTGACGG
Nqo1-Forward	GAAGACATCATTCAACTACGCC
Nqo1-Reverse	GAGATGACTCGGAAGGATACTG
Gsta4-Forward	AGTACCCTTGGTTGAAATCGAT
Gsta4-Reverse	GGTCCTTCCCATACAAGTTGTA

*Primers were designed and synthesized by Sangon Biotech (Shanghai, China)*.

### Western Blotting Results

**Figure 5:**
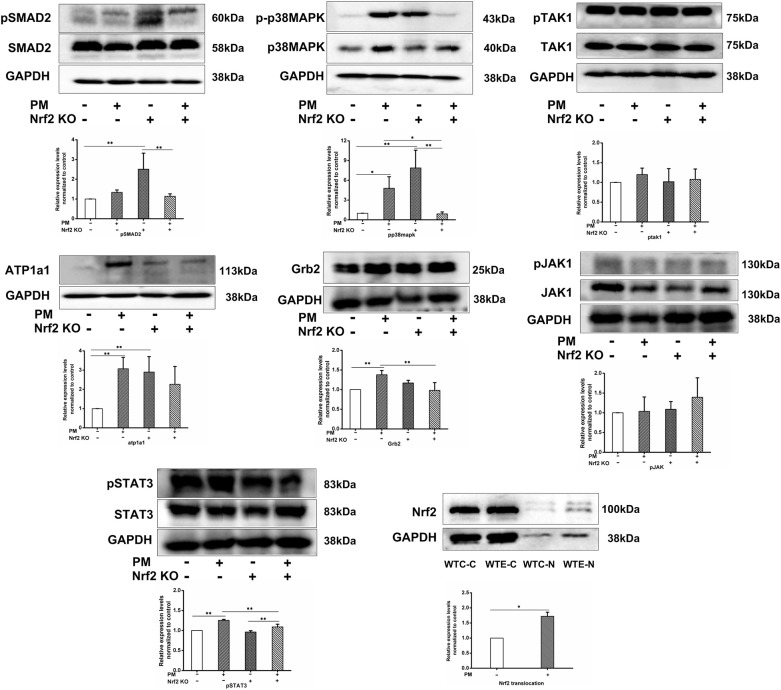
Western blotting results. For 6 weeks, 6–8 weeks C57/B6 mice, with or without Nrf2 knockout, were exposed to either filtered air or air containing particulate matter (PM), and then the hearts were collected, snap-frozen in liquid nitrogen, extracted, and subjected to western blotting for p-SMAD2, SMAD2, p-p38MAPK, p38MAPK, p-TAK1, TAK1, ATP1a1, Grb2, p-JAK1, JAK1, p-STAT3, STAT3, and GAPDH. An additional group of WTC and WTE heart samples was processed with nuclear and cytoplasmic extraction kit (Epizyme, Shanghai, China) and probed for Nrf2 and GAPDH. *N* = 3 per group from three independent experiments. *Statistically different between the two groups (*P* < 0.05). **Statistically different between the two groups (*P* < 0.01).

To further verify the changes of the TGF-β and MAPK signaling pathways at the translational and post-translational levels, western blotting was performed on the heart protein samples for phosphorylated SMAD2, SMAD2, phosphorylated TAK1, TAK1, phosphorylated MAPK, MAPK, ATP1A1, Grb2, phosphorylated JAK1, JAK1, phosphorylated STAT3, and STAT3. To confirm the activation level of Nrf2, additional batches of samples were processed with the nuclear and cytoplasmic extraction kit (Epizyme, Shanghai, China), and then western blotting was performed for Nrf2 in both the cytoplasmic and nuclear protein of same samples. The results (Figure 5) indicated that the phosphorylated SMAD2 levels were significantly increased by Nrf2 knockout, but they were also effectively decreased by PM exposure; PM exposure remarkably elevated the phosphorylation levels of p38MAPK, while Nrf2 knockout enhanced the phosphorylation even further. Interestingly, co-treatment of Nrf2 knockout and PM exposure lead to abolishment of the p38MAPK phosphorylation. Additionally, no significant changes in the phosphorylation of TAK1 were detected. ATP1A1 expression levels were found to be remarkably elevated by either PM exposure or Nrf2 knockout, but, interestingly, co-treatment with both PM exposure and Nrf2 knockout lead to lower expression levels. Nrf2 nuclear translocation was found to be significantly elevated by PM exposure. While no significant changes were detected in phosphorylated JAK1 levels, elevated Grb2 and phosphorylated STAT3 expression levels were observed following PM exposure, which were effectively decreased in Nrf2 knockout animals comparing to wildtype counterparts.

### Mortality Analysis and MDA Results

**Figure 6:**
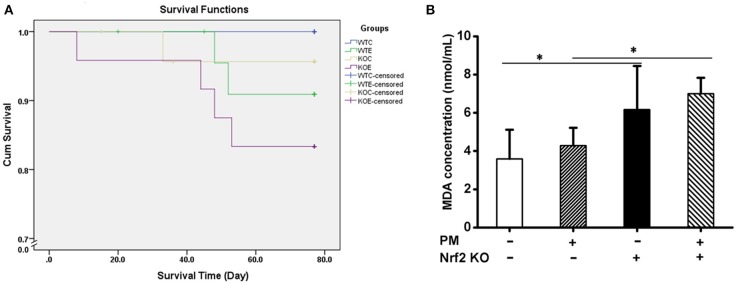
Survival Plot and MDA contents in serum. For 11 weeks, 6–8-weeks C57/B6 mice, with or without Nrf2 knockout, were exposed to either filtered air or air containing particulate matter (PM), during which the dead animals were recorded, and the Kaplan-Meier survival plot was made. Serum from animals exposed to PM for 6 weeks were subjected to MDA assay with a commercially available kit (Solarbio, China, BC0025). *N* = 4 per group. *Statistically different between the two groups (*P* < 0.05). **(A)** Kaplan-Meier survival plot of the animals. **(B)** MDA assay results of the animal serums.

The Kaplan-Meier survival plot of the animals is presented in Figure 6A. No statistical differences were observed among the groups. The MDA contents in the serum of mouse exposed to PM for 6 weeks are reported in Figure 6B. Significantly elevated MDA levels were observed in the two KO groups relative to their corresponding wildtype controls.

## Discussion

This study was performed along with Li et al.'s study (Li et al., 2019), which assessed multiple organs, including the lung, brain, heart, testis, and intestine. While similar changes were revealed in that study, the current study has focused on cardiovascular effects and has investigated more extensively the underlying molecular mechanisms of Nrf2-knockout animal and RNA-seq techniques. In this study, the most relevant exposure to PM was achieved with the IVC real-ambient exposure system, which helped to determine the subacute cardiovascular effects of PM exposure at real-life levels in Shijiazhuang, China. Meanwhile, the utilization of Nrf2-knockout mice helped to determine whether the major antioxidant gene Nrf2 plays a role in PM-mediated toxicities.

It has been generally accepted that PM exposure increases cardiovascular-related risks in sensitive populations (Slaughter et al., 2005; Dominici et al., 2006; Mieczyslaw, 2008; Santos et al., 2008; Chiusolo et al., 2011; Rajagopalan et al., 2018). For example, cardiovascular-related mortality was reported to be increased in the Chinese population following PM exposure (Haidong et al., 2007; Yanjun et al., 2011; Zhang C. et al., 2018). Also, data from Europe and America with lower levels of PM2.5, where the range of PM2.5 exposure was lower than 35 μg/m^3^ (6.7–25 μg/m^3^), also showed that the increase of PM2.5 levels can lead to an increase in the risk of cardiovascular mortality (Bart et al., 2006; Antonella and Joel, 2009; Kadri et al., 2012; Talbott et al., 2014). Additionally, an increased incidence of cardiovascular events was associated with increased PM exposure as well (Lawal, 2017). In laboratory studies, it is common to see that intratracheal dripping with PM particles leads to negative effects in the cardiovascular system regarding activities such as cardiac function, blood pressure, and cardiomyopathy (Wang H. et al., 2017). In the current study, PM exposure was found to increased heart rate after 6 weeks of exposure, and this was consistent with Bennett et al.'s study (Bennett et al., 2018) in which elevated heart rate was reported following PM exposure. Additionally, significant decreases in other functional parameters, including decreased stroke volume, ejection fraction, cardiac output, and fractional shortening were also observed. Interestingly, many of these parameters exhibited similar patterns: 6-weeks PM exposure led to negative changes, but 11-weeks exposure led to somewhat alleviated changes. This might be explained by the higher mortality in animals exposed to PM for 11 weeks, as indicated by our mortality data. Histological assessments revealed a unique pathological change in the right ventricular wall: the PM exposure seemed to have thickened the right ventricular wall in both wildtype and knockout animals. The thickening/thinning of the right ventricular wall is a mark of cardiac remodeling (Pape et al., 2002), and thus this endpoint suggests the risk of cardiac remodeling following PM exposure. Cardiac remodeling, while is associated with air pollution itself (Minicucci et al., 2009), significantly contributes to common cardiovascular conditions, such as heart failure (Chaanine, 2019) and myocardial infraction (Jia et al., 2014). The potential association between PM exposure and cardiac remodeling might partially explain the observed elevated incidence of cardiovascular events following high PM exposure (Dominici et al., 2006). However, high magnitude pictures indicated no significant change in the cross-section area of cardiomyocytes (data not shown), which is probably due to the relatively short exposure periods. A longer exposure period is needed to further elucidate such changes in the right ventricular wall.

The composition of air pollutants is complex and contains a variety of metal elements. Heavy metals, as the main inorganic components in PM10 and PM2.5, are known for remarkable bioaccumulation (Li et al., 2015). A common method to determine the bioaccumulation of heavy metals is ICP-MS, which features high sensitivity, a low detection limit, fast analysis speed, and public acceptance in the area of heavy metals detection in atmospheric particles (Ohata and Nishiguchi, 2018). It had been reported that PM particles are capable of promoting heavy metal deposition in the heart (Ku et al., 2017). In the current study, the contents of metals (Na, Mg, Ni, Cu, Al, K, Zn, Se, Ca, Cr, Sr, Ba, Mn, Fe, and Pb) in cardiac tissues of mice exposed to PM for 11 weeks were determined with ICP-MS. The results were consistent with previous reports, and indicated that the levels of Na, K, Se, and Fe in the cardiac tissues of mice in the PM exposure group increased significantly, suggesting a remarkable role of PM exposure in the deposition of heavy metals in heart tissues (Feng et al., 2017).

To evaluate the molecular mechanisms by PM-induced cardiotoxicity, high-throughput RNA sequencing (RNA-seq) of the expression level of all genes in the cardiac tissue was performed. RNA-seq features high throughput screening for differentially expressed genes (DEGs) and is capable of detection of DEGs following exposure to a variety of toxicants, such as perfluorooctanoic acid (Sheng et al., 2018) and 2,3,7,8-Tetrachlorodibenzodioxin (Lai et al., 2018). In the current study, PM exposure resulted in over 2,000 DEGs in mice hearts relative to the control in the current study, suggesting a wide range of effects. KEGG enrichment analysis revealed several signaling pathways most likely being affected by PM exposure, including the MAPK pathway, the phagosome pathway, the calcium signaling pathway, the Ras signaling pathway, the TGF-βsignaling pathway, the oxidative phosphorylation, and the insulin signaling pathway. Among these potential targets, the MAPK signaling pathway and the TGF-βpathway have frequently been associated with cardiotoxicity in previous studies (Cao et al., 2016; Wang J. et al., 2017; Zhang Y. et al., 2018), and thus they were selected and pursued further with qRT-PCR and western blotting.

Nrf2 is one of the antioxidant genes that received high level of attentions. While Nrf2-mediated cardiac protection has commonly been reported in various studies (Deng et al., 2013; Smith et al., 2016; Erkens et al., 2018), the mixed roles of Nrf2 in various pathogenesis process and inflammation have also been reported (Satta et al., 2017; da Costa et al., 2019; Pompili et al., 2019). Specifically, negative effects of Nrf2 in cardiovascular diseases were reported (Barajas et al., 2011; Harada et al., 2012). Particulate matter exposure was also associated with elevated expression of Nrf2, which was abolished by protections (Guan et al., 2019), suggesting that Nrf2 indeed plays roles in PM-induced cardiotoxicity, but this role is not necessarily purely beneficial or purely detrimental. In the current study, it has been revealed that Nrf2 nuclear translocation indeed increased following PM exposure, suggesting activation of this signaling pathway in response to PM exposure. However, in contrary to our expectation, the Nrf2 knockout animals had a mixed response following PM exposure comparing to their wildtype littermates: Nrf2 knockout indeed further deteriorated some of the endpoints, such as the left ventricle mass and diastolic left ventricular volume. Increased left ventricle mass indicated the potential of cardiac hypertrophy, which has commonly been reported following Nrf2 knockout (Shanmugam et al., 2017). On the other hand, the knockout of Nrf2 abolished some of the detrimental changes observed in wildtype exposed animals, such as stroke volume, ejection fraction, fractional shortening, and systolic left ventricular volume. Notably, lower baselines of these functional parameters were present for the knockout animals, which is consistent with Shanmugam et al. (2017). While no definitive explanations are available at this point, it may be associated with Nrf2's antioxidant properties. Similar effects were also observed in the changes of the signaling molecules, such as AKT1, TAK1, DDIT3, PPP2CB, ABCA1, BMP2, ATP1A1, Dag1, and Grb2 and phosphorylation of p38MAPK/STAT3. Taken these facts together, it is suggested that Nrf2 may partially participate in the PM-induced cardiotoxicity instead of exerting complete protective roles as expected. In summary, Nrf2 seems to play a mixed role in PM-induced cardiotoxicity, exerting some detrimental effects as well as some protective effects. Further studies are needed regarding to the exact molecular mechanism for this phenomenon.

The TGF-βsignaling pathway is involved in inflammation and fibrosis (Bonay et al., 2015), thus participating in cardiac remodeling (Zhang Y. et al., 2018). Qin et al. (2018) reported PM exposure induced TGF-β signaling pathway activation following 3 mg/kg oropharyngeal aspiration for 4 weeks. In the current study, lower exposure doses (estimated cumulative dose of 0.28–0.35 mg/kg) and longer exposure duration (up to 11 weeks) provided more realistic assessments: both RNA-seq and qRT-PCR results revealed that TGF-β1 expression levels in the heart were slightly suppressed following PM exposure and/or Nrf2 knockout. Furthermore, western blotting revealed that the phosphorylation of SMAD2 in wildtype animals exposed to PM had an increasing trend but was not statistically significant. On the other hand, as a related signaling molecule, BMP2 levels were significantly increased in PM-exposed wildtype animals. BMP2 is involved in cardiac remodeling as well (Saxon et al., 2017). Taking the data together, it seems like PM exposure had limited effects on TGF-β1, while BMP2 seems to be a target of PM exposure. Interestingly, significantly decreased TGF-β1 expression levels were observed in Nrf2 knockout animals exposed to PM compared to Nrf2 knockout animals without PM exposure. This phenomenon will be investigated in future studies. Moreover, the magnitude of changes in the expression levels of signaling molecules seemed to be larger than those in morphological and functional changes, which is probably due to the longer time required for prominent morphological and functional changes to emerge.

The MAPK signaling pathway is widely involved in proliferation/cell survival processes, such as tumor growth and cardiac remodeling. As indicated in the RNA-seq results, MAPK seems to be involved in PM-induced cardiotoxicity. We further investigated affected molecules, primarily p38MAPK, TAK1, and the related JAK-STAT signaling pathway. The JAK-STAT signaling pathway participates in inflammatory responses and cardiomyocyte proliferation (Hashmi et al., 2019; Jamilloux et al., 2019), which had been reported to be activated by MAPK signaling (Popielarczyk et al., 2019; Wang et al., 2019). PCR results indicated that the expression levels of TAK1, JAK, STAT3, and Grb2 all remarkably increased following PM exposure, while Nrf2 knockout seems to have reversed the changes. Meanwhile, the increased expression of p38MAPK was only observed in Nrf2 knockout animals without PM exposure. Western blotting results indicated that, although the TAK1 and JAK mRNA levels seemed to be altered following PM exposure and/or Nrf2 knockout, the phosphorylation level of the corresponding proteins displayed no significant changes. On the other hand, the phosphorylation level of p38MAPK and STAT3 effectively increased in PM-exposed wildtype animals, while the Nrf2 knockout seemed to abolish such effects. These results are consistent with Cao et al.'s study (Cao et al., 2016) in which PM exposure activated p38MAPK in H9c2 cells. The abolishment of p38MAPK and STAT3 phosphorylation suggested that the activation of p38MAPK and JAK-STAT was at least partially mediated through Nrf2, which has frequently been reported to interact with p38MAPK (Wang J. et al., 2017). Our data suggest that p38MAPK seems to be one of the mechanisms contributing to PM-induced cardiotoxicity located downstream of Nrf2.

In the current study, PM-induced cardiotoxicity and the roles of Nrf2 were investigated. While several findings were discovered, potential pitfalls exist, and attention is needed while interpreting the data. There are two major points of concern. The first one is the increased mortality in knockout animals, especially in those exposed to PM for 11 weeks. This might be the result of increased oxidative stress, as indicated by the results of the MDA assay. The death of sensitive animals might have contributed to the observed abolishment of PM-induced cardiotoxicity. The second point of concern is the signaling pathway changes in knockout animals without PM exposure. The data suggested that this group of animals have some quite notable signaling changes compared to the wildtype control animals, such as the phosphorylation of SMAD2 and p38MAPK. These might be explained by the fact that the TGF-β and MAPK signaling pathways both interact with Nrf2 (Liu J. et al., 2018), but such effects definitely added uncertainty to this study.

## Conclusions

In summary, PM-induced cardiac morphology and functional changes were observed in the current study, suggesting that the real-ambient level of PM exposure may indeed induce cardiotoxicity in C57/B6 mice. The knockout of Nrf2 lead to alleviation of several cardiac parameters as well as reversion of molecular signaling changes, suggesting that Nrf2 plays a role in PM-induced cardiotoxicity. Notably, Nrf2 knockout itself altered the baseline of morphological and functional parameters of the heart without PM exposure. This result is in opposition to the classical protective role of Nrf2 and is worthy further investigation.

## Data Availability Statement

The materials described in the article, including all relevant raw data, will be freely available to any scientist wishing to use them for non-commercial purposes, and the data used in the current study can be obtained from the corresponding authors on a reasonable request.

## Ethics Statement

All the procedures used in this study have been approved by the Qingdao University Animal Care and Use Committee in keeping with the National Institutes of Health guidelines.

## Author Contributions

YZ and RZ designed the study and revised the manuscript. LCu, DL, and QJ performed the data analysis and drafted the manuscript. JP, RC, and WC assisted the manuscript writing. LCu, AJ, and LS performed the statistical analysis. LS, LCu, LCh, MJ, AJ, XS, and XL carried out animal examinations and collected data. QJ, JW, JT, LS, and AJ performed the histopathology experiments. JW and CC performed RNA-seq data analysis. All authors read and approved the final manuscript.

### Conflict of Interest

The authors declare that the research was conducted in the absence of any commercial or financial relationships that could be construed as a potential conflict of interest.
