# Supplementary material for: Real-Ambient Particulate Matter Exposure-Induced Cardiotoxicity in C57/B6 Mice
Source: Front Pharmacol. 2020 Mar 31;11:199. doi: 10.3389/fphar.2020.00199 (PMC7136766; doi:10.3389/fphar.2020.00199)
Supplement: Supplementary file 1 [file Table_1.DOCX]

Supplementary Table 1 Heavy metal element content(mg/kg) in knockout animals

|  | KOC | KOE | p |
| --- | --- | --- | --- |
| Na | 1605.76±109.11 | 1533.94±50.06 | 0.27 |
| Mg | 171.43±7.12 | 186.53±15.02 | 0.12 |
| Ni | 0.04±0.01^b^ | 0.07±0.02 | 0.10 |
| Cu | 6.02±0.35 | 6.14±7.29 | 0.78 |
| Al | 28.94±11.73 | 27.04±7.29 | 0.79 |
| K | 2212.38±113.23 | 2483.81±190.78 | 0.05* |
| Zn | 19.91±1.82 | 22.52±2.15 | 0.11 |
| Se | 0.23±0.06 | 0.36±0.06 | 0.02* |
| Ca | 40.28±8.23 | 42.01±5.11 | 0.73 |
| Cr | 0.45±0.16 | 1.12±0.33 | 0.01* |
| Sr | 0.35±0.08 | 0.39±0.11 | 0.53 |
| Ba | 0.74±0.20 | 0.94±0.30 | 0.30 |
| Mn | 0.56±0.09 | 0.67±0.04 | 0.06 |
| Fe | 78.64±12.60 | 100.16±9.57 | 0.04* |
| Pb | 0.14±0.03 | 0.21±0.06 | 0.07 |

KOC, knock out control; KOE, knock out exposure.

Data are means ± SD (n = 16).
