# Supplementary material for: Real-Ambient Particulate Matter Exposure-Induced Cardiotoxicity in C57/B6 Mice
Source: Front Pharmacol. 2020 Mar 31;11:199. doi: 10.3389/fphar.2020.00199 (PMC7136766; doi:10.3389/fphar.2020.00199)
Supplement: Supplementary file 2 [file Table_2.DOCX]

Supplementary Table 2 Lists of most upregulated and downregulated genes from the RNA-seq results.

1. KOC vs KOE (up gene)

| **Gene name** | **KOC** | **KOE** | ***p* −value** |
| --- | --- | --- | --- |
| Nepn | 31.50033 | 90.17724 | 4.44E-07 |
| Pdgfb | 2692.057 | 4532.894 | 8.01E-07 |
| Tcf15 | 1320.817 | 2406.007 | 3.43E-06 |
| Galnt18 | 736.423 | 1067.921 | 5.34E-06 |
| Timeless | 471.0865 | 854.0573 | 6.91E-06 |
| Alb | 14.03643 | 261.7174 | 1.80E-05 |
| Mup7 | 0.295887 | 22.88515 | 2.28E-05 |
| Kdr | 6379.892 | 9505.342 | 4.16E-05 |
| Sema6a | 482.4198 | 752.4369 | 7.48E-05 |
| Gm13889 | 281.547 | 419.9369 | 8.86E-05 |
| Afdn | 1738.991 | 2733.062 | 8.99E-05 |
| Ets2 | 2248.896 | 3812.143 | 9.78E-05 |
| Slfn3 | 81.91135 | 154.7672 | 9.80E-05 |
| Tpst1 | 794.2955 | 1098.269 | 0.000156669 |
| Fgg | 0.430023 | 18.92785 | 0.000164503 |
| Nos2 | 232.9696 | 480.7989 | 0.000188093 |
| Gc | 3.634647 | 41.16217 | 0.000189374 |
| Apbb2 | 810.7238 | 1102.007 | 0.000214236 |
| Sema4c | 611.8278 | 950.8225 | 0.000221782 |
| Serpina1e | 5.097942 | 51.11445 | 0.000232917 |

1. KOC vs KOE (down gene).

| **Gene name** | **KOC** | **KOE** | ***p* −value** |
| --- | --- | --- | --- |
| Nrgn | 1127.992 | 178.0824 | 2.74E-12 |
| Gp9 | 131.6961 | 12.58807 | 4.72E-09 |
| Tmem40 | 34.12712 | 2.347312 | 8.67E-09 |
| Gnao1 | 964.4872 | 383.3491 | 9.63E-09 |
| Ppbp | 371.2881 | 48.73325 | 1.51E-08 |
| Itga2b | 426.3212 | 82.32154 | 4.64E-08 |
| Treml1 | 78.20448 | 8.2673 | 5.20E-08 |
| Tubb1 | 192.9852 | 22.48545 | 5.68E-08 |
| Cpne5 | 140.9835 | 14.85035 | 9.28E-08 |
| Gm6634 | 67.21657 | 8.105789 | 1.54E-07 |
| Clec1b | 157.8593 | 26.58752 | 1.57E-07 |
| Ryr3 | 108.6742 | 14.88523 | 1.62E-07 |
| Treml2 | 73.40718 | 21.83462 | 3.22E-07 |
| Pam | 26389.42 | 8377.81 | 3.45E-07 |
| Cd226 | 36.03269 | 8.626618 | 5.43E-07 |
| Rgs18 | 79.81916 | 16.00786 | 7.07E-07 |
| F5 | 84.48639 | 12.7151 | 8.58E-07 |
| Krt80 | 114.8788 | 56.7391 | 1.01E-06 |
| Igsf23 | 189.6854 | 48.71476 | 1.21E-06 |
| Adcy1 | 202.1366 | 48.90785 | 1.80E-06 |

1. WTC vs WTE (up gene).

| **Gene name** | **WTC** | **WTE** | ***p* −value** |
| --- | --- | --- | --- |
| Ucp3 | 562.8903 | 2482.452 | 2.67E-08 |
| Prdm11 | 279.318 | 442.0745 | 4.52E-08 |
| Slc7a1 | 1005.581 | 2031.343 | 5.44E-08 |
| Slc25a22 | 823.9951 | 1584.839 | 1.10E-07 |
| Slc25a42 | 1077.061 | 2074.839 | 1.20E-07 |
| Synpo | 2932.594 | 4793.682 | 1.70E-07 |
| Dusp18 | 536.6901 | 1403.326 | 2.64E-07 |
| Mlycd | 3398.502 | 5098.927 | 3.39E-07 |
| Alpk2 | 1846.182 | 3354.892 | 7.55E-07 |
| Ehhadh | 232.1751 | 504.0579 | 8.18E-07 |
| Prune1 | 456.6085 | 711.097 | 8.96E-07 |
| Fosl2 | 1067.413 | 2106.396 | 1.23E-06 |
| Fitm2 | 4032.503 | 6213.044 | 1.31E-06 |
| Dmpk | 12421.77 | 17254.72 | 2.74E-06 |
| Sik1 | 850.421 | 2336.63 | 3.35E-06 |
| Gpr146 | 1982.478 | 2722.011 | 3.67E-06 |
| Popdc3 | 565.034 | 866.5068 | 4.55E-06 |
| Kremen1 | 2629.293 | 4440.705 | 5.27E-06 |
| Trmt5 | 521.7321 | 726.315 | 1.05E-05 |
| Pcdhga2 | 32.11231 | 70.75163 | 1.26E-05 |

1. WTC vs WTE (down gene).

| **Gene name** | **WTC** | **WTE** | ***p* −value** |
| --- | --- | --- | --- |
| Gm26917 | 2430.988 | 1258.938 | 5.06E-18 |
| Rps27a | 4199.956 | 1612.241 | 4.12E-12 |
| Ckb | 2869.235 | 1772.551 | 2.36E-10 |
| Ddit3 | 861.7646 | 450.6535 | 9.76E-10 |
| Rarg | 1670.346 | 914.3135 | 3.04E-08 |
| Gbp2 | 1049.737 | 619.1369 | 2.01E-07 |
| Lyl1 | 566.0987 | 285.0446 | 8.41E-07 |
| Arrb2 | 785.3745 | 516.8513 | 1.49E-06 |
| Banp | 419.5122 | 184.2035 | 3.27E-06 |
| Mir99ahg | 354.2792 | 174.9219 | 4.47E-06 |
| Apol9b | 115.2344 | 58.18801 | 1.23E-05 |
| Pdgfa | 544.8763 | 396.6495 | 1.40E-05 |
| Lfng | 831.6796 | 437.0528 | 1.73E-05 |
| H2-T23 | 4078.453 | 2462.226 | 2.17E-05 |
| Psmb8 | 1672.202 | 917.7396 | 2.32E-05 |
| Gm13123 | 83.54581 | 28.86907 | 2.76E-05 |
| S100a13 | 1516.861 | 893.8462 | 2.77E-05 |
| Rpl3-ps1 | 652.797 | 401.6332 | 3.10E-05 |
| Aif1 | 217.7153 | 113.071 | 3.14E-05 |
| Med20 | 455.3835 | 310.1241 | 3.46E-05 |
