# Supplementary material for: Real-Ambient Particulate Matter Exposure-Induced Cardiotoxicity in C57/B6 Mice
Source: Front Pharmacol. 2020 Mar 31;11:199. doi: 10.3389/fphar.2020.00199 (PMC7136766; doi:10.3389/fphar.2020.00199)
Supplement: Supplementary file 3 [file Data_Sheet_1.docx]

**Supplementary Figure Legend**

**Supplementary Figure 1 Confirmation of Nrf2 knockout**

The Nrf2 knockout mouse line were confirmed with DNA extraction and electrophoresis in agarose gel. The Nrf2 knockout mice will have a 750 bp band, while the wildtype mice will have a 400 bp band, as indicated in the figure.

**Supplementary Figure 2 Representative echocardiography images**

Representative echocardiography images are shown. WTC: wildtype control; WTE: wildtype exposure; KOC: knockout control; KOE: knockout exposure.

**Supplementary Figure 3 Measurement method of right ventricular wall thickness**

In adobe photoshop 7.0, two rulers were applied to the heart picture as shown in the figure. The first ruler has seven measurement lines evenly distributed, with 22.5-degree angle in between. The size and orientation of the ruler was adjusted so that the two ends sit on the two ends of the free right ventricular wall. The second ruler was then used to make sure the measurement is perpendicular to the ventricular wall. Star indicates the measurement points for the specific site.
