# Supplementary figures and images for: Real-Ambient Particulate Matter Exposure-Induced Cardiotoxicity in C57/B6 Mice
Source: Front Pharmacol. 2020 Mar 31;11:199. doi: 10.3389/fphar.2020.00199 (PMC7136766; doi:10.3389/fphar.2020.00199)

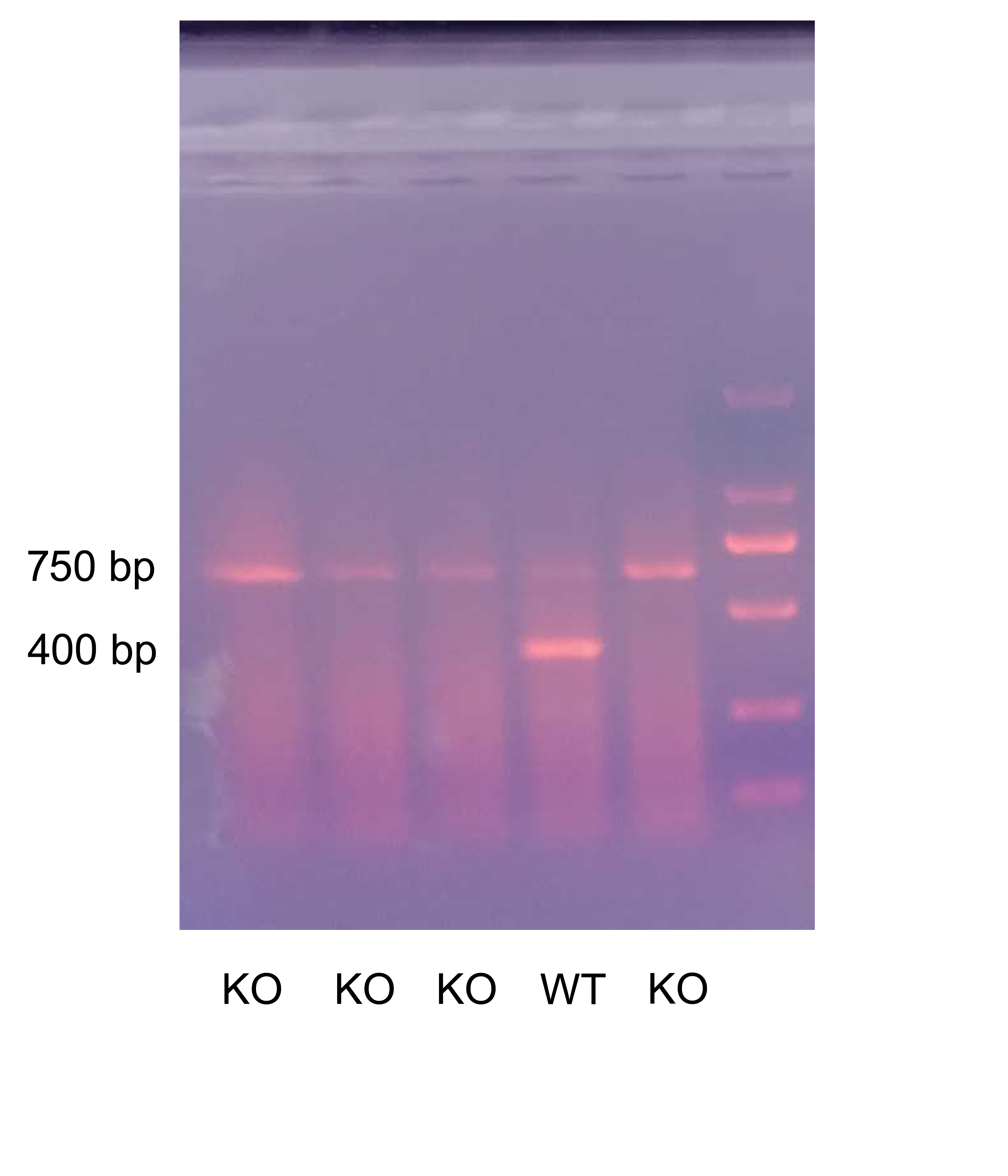

Supplement: Supplementary file 4 [file Image_1.TIF]

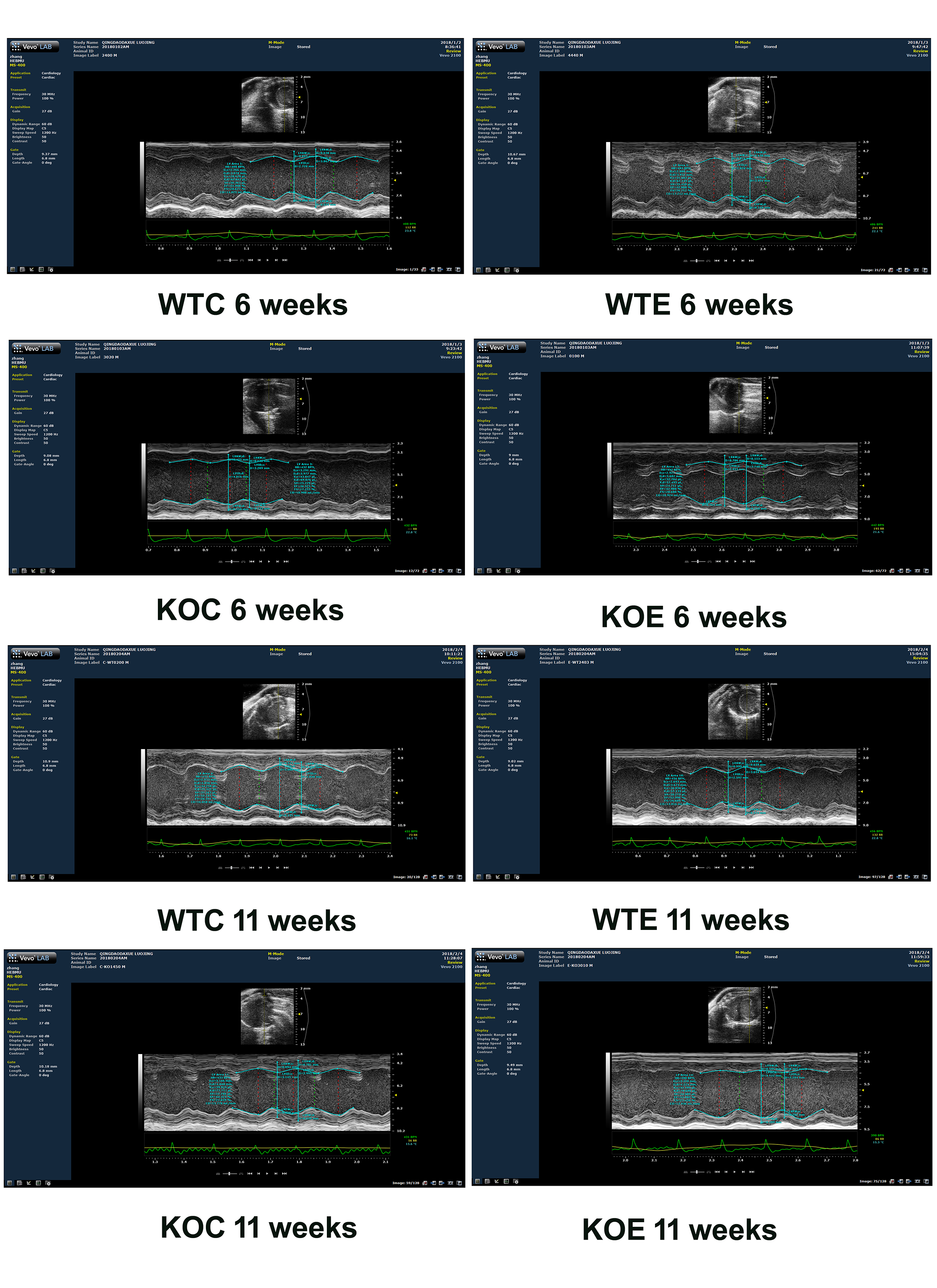

Supplement: Supplementary file 5 [file Image_2.TIF]

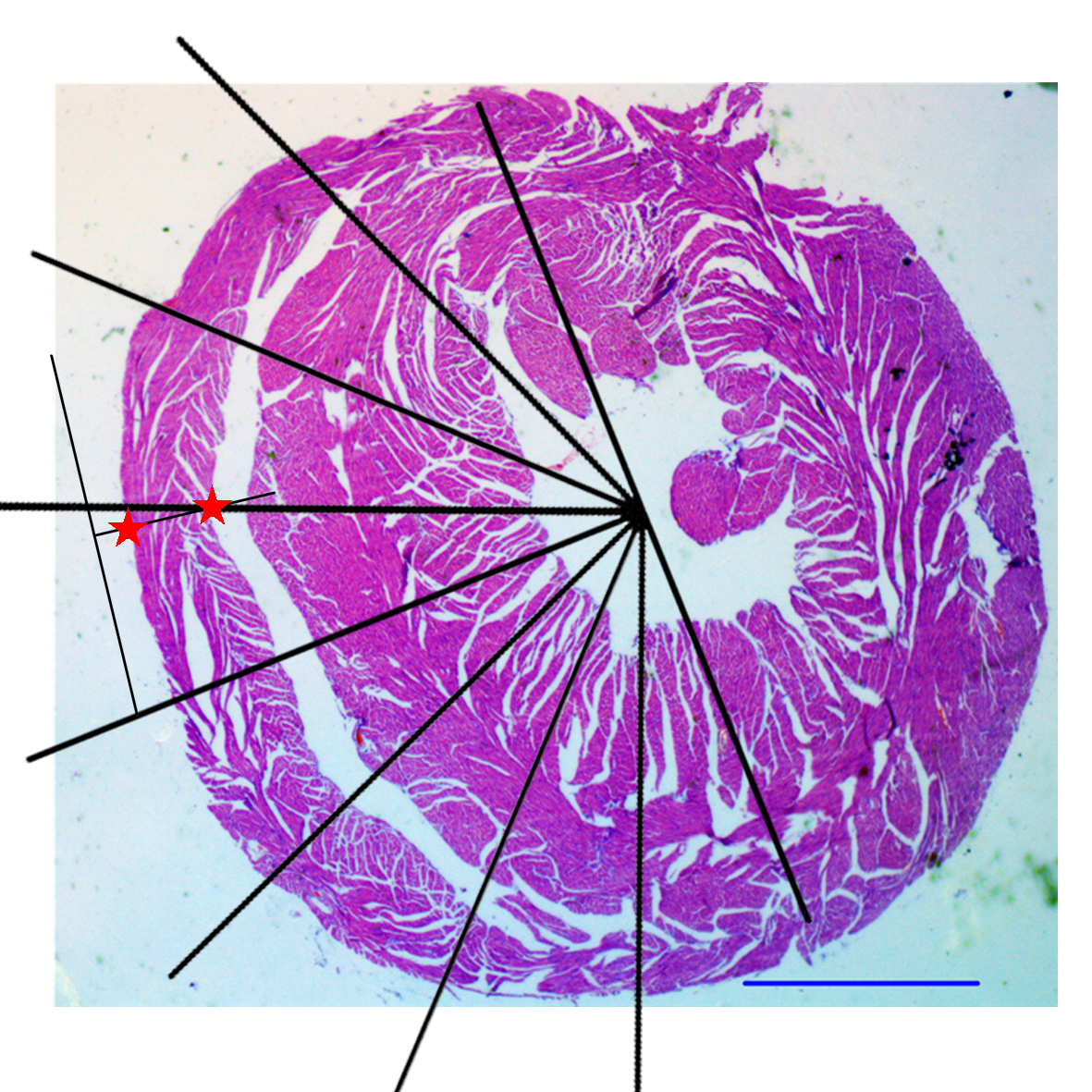

Supplement: Supplementary file 6 [file Image_3.TIF]
